# Electrochemical Comparison of SAN/PANI/FLG and ZnO/GO Coated Cast Iron Subject to Corrosive Environments

**DOI:** 10.3390/ma11112239

**Published:** 2018-11-11

**Authors:** Muhammad Khitab Ahmed, Muhammad Shahid, Zulfiqar Ahmad Khan, Ameen Uddin Ammar, Abdul Saboor, Amir Khalid, Asad Hayat, Adil Saeed, Mehran Koohgilani

**Affiliations:** 1 School of Chemical & Material Engineering (SCME), National University of Engineering & Technology (NUST), Islamabad 46000, Pakistan; khitab.770@gmail.com (M.K.A.); mshahid@scme.nust.edu.pk (M.S.); amir_ms08@scme.nust.edu.pk (A.U.A.); saboorab@gmail.com (A.S.); ameenuddin03@gmail.com (A.K.); asadhanjra@gmail.com (A.H.); 2 NanoCorr, Energy & Modelling (NCEM) Research Group, Department of Design & Engineering, Bournemouth University, Poole BH12 5BB, UK; asaeed4@bournemouth.ac.uk (A.S.); mkoohgilani@bournemouth.ac.uk (M.K.); 3 Renewable Energy Engineering (REE), U.S-Pakistan Center for Advance Studies in Energy (USPCAS-E), University of Engineering and Technology Peshawar, Khyber Pakhtunkhwa 25120, Pakistan

**Keywords:** nanocomposite coating, polyaniline, graphene oxide (GO), zinc oxide, styrene acrylonitrile, few layers graphene, electrochemical impedance spectroscopy

## Abstract

ZnO/GO (Graphene Oxide) and SAN (Styrene Acrylonitrile)/PANI (Polyaniline)/FLG (Few Layers Graphene) nanocomposite coatings were produced by solution casting and sol-gel methods, respectively, to enhance corrosion resistance of ferrous based materials. Corrosive seawater and ‘produced crude oil water’ environments were selected as electrolytes for this study. Impedance and coating capacitance values obtained from Electrochemical Impedance Spectroscopy (EIS) Alternating Current (AC technique) showed enhanced corrosion resistance of nanocomposites coatings in the corrosive environments. Tafel scan Direct Current (DC technique) was used to find the corrosion rate of nanocomposite coating. SAN/PANI/FLG coating reduced the corrosion of bare metal up to 90% in seawater whereas ZnO/GO suppressed the corrosion up to 75% having the impedance value of 100 Ω. In produced water of crude oil, SAN/PANI/FLG reduced the corrosion up to 95% while ZnO/GO suppressed the corrosion up to 10%. Hybrid composites of SAN/PANI/FLG coatings have demonstrated better performances compared to ZnO/GO in the corrosive environments under investigation. This study provides fabrication of state-of-the-art novel anti corrosive nanocomposite coatings for a wide range of industrial applications. Reduced corrosion will result in increased service lifetime, durability and reliability of components and system and will in turn lead to significant cost savings.

## 1. Introduction

Corrosion is an electrochemical phenomenon which leads to severe component, system and structural deteriorations. Various operational conditions lead to corresponding corrosion failure mechanisms such as pitting, crevice, uniform, galvanic and microbial corrosion. Combined with static or dynamic stresses corrosion often leads to corrosion fatigue, fretting and stress corrosion cracking. These damages cause replacement, unscheduled maintenance, reduced capacity, idle time and insurance issues. Various techniques are used to control corrosion, including design modification, inhibitors, cathodic protection, thereby, increasing the cost of maintaining operations. Corrosion also has several economic, health, safety and technological implications [1]. Many industries face huge financial losses due to corrosion-led failures. Researchers have been developing dielectric materials with enhanced corrosion resistance capabilities to both avoid and mitigate corrosion failures within structures and interacting systems. Some researchers have found that hybrid organic and/or inorganic nanocomposite materials are becoming desirable candidate to perform well in corrosive applications.

Few Layers Graphene (FLG) with 5–8 layers of graphene exhibit the promising properties of thermal, chemical and mechanical stability and are also a good charge carrier; this makes them fascinating in various fields of application [2,3,4]. Graphene provides resistance against oxidation while its hydrophobicity resists hydrogen bonding with water. Due to its multiple promising properties, graphene is being extensively explored for various applications including interacting systems and as nano-additives in lubrication. Recently, several studies have reported that graphene coating can effectively isolate the surface from environmental degradation [5,6,7]. Aneja et al. [4] studied functionalized graphene coating to evaluate its barrier and protection properties applied to mild steel.

Water uptake of the coating, its capacitance and pore resistance was obtained by using EIS. Experiments were conducted at various frequencies for a certain time period. Graphene decreased the water uptake and the activation energy peak was increased for water driven diffusivity process constraining the ion movement through the coating. Moreover, the network of functionalized graphene was found to be structurally dense [4]. Similarly, Olad et al. [8] investigated a triple hybrid system of conducting polyaniline, zinc and epoxy to evaluate their corrosion resistant properties when applied to metallic substrates. During this research, 75-micron thick nanocomposite coatings on iron substrate and in a solution of 0.1 M HCl solution were studied. In addition to anti corrosive properties, epoxy and zinc enhanced the mechanical strength of these nanocomposite coatings. Epoxy enhanced the mechanical properties by providing physical hindrance to electrolyte molecules while zinc improved the electrochemical behavior of polyaniline coating.

SAN is copolymer of styrene and acrylonitrile. It helps against corrosion degradation facilitated by its hydrophobicity and provides physical barrier, based on higher diffusion resistance to the corrosive ion [9]. Conducting polymer prevents corrosion with the help of generated electric field that blocks the electrons flow from the metal to external oxidizing agents [10]. Polyaniline is one of the promising polymers for corrosion protection. It is a p-type semiconductor with desirable electrical and chemical properties. Its conductive type contributes significantly to the effective resistance to corrosion.

New trends in the processing of Polyaniline have increased its range of practical applications. It protects against electrochemical decay by forming a thin oxide layer due to the interaction of polymer and metal substrate. It reduces the redox current as well as shifts the corrosion potential towards noble direction as compared to bare metal [11]. ZnO is a wide bandgap inorganic conductor. It belongs to II-VI semiconductor family. It is found in the form of a white colored powder. Its hydrophobicity and ability to shift corrosion potential to noble direction makes it a good candidate for corrosion protection [12,13,14].

In this research novel hybrid nanocomposite coatings of SAN/PANI/FLG and ZnO/GO were synthesized and characterized through various characterizing tools. An electrochemical framework of Gamry^®^ Instruments was used to evaluate the performances of coating systems against corrosion to protect ductile cast iron pipeline in seawater and crude oil produced water. Electrochemical Impedance Spectroscopy (EIS) technique employs a short perturbation AC signals during electrochemical investigation [15,16,17,18,19,20]. Amplitude of 10 mV AC was applied to investigate the response of system under various frequencies. Corrosion resistance and protection are analyzed by plots Bode and Nyquist generated by EIS. Phase shift and impedance values are plotted against frequency in Bode plot while imaginary frequency is scanned versus real impedance in Nyquist plot. SAN/PANI/FLG performed better in protecting metal samples in aggressive environments against corrosion compared to ceramic ZnO/GO nanocomposite coating.

## 2. Materials and Methods 

### 2.1. Sample Preparation

Ductile cast iron industrial pipeline samples were used as substrates. Samples were prepared in ~4 × 4 cm^2^ squared sections. Samples were ground with abrasive paper of 120–320 grades in order to develop a texture for adhesion of nanocomposites coating. Table 1 shows composition of the pipeline cast iron used for study.

### 2.2. Synthesis of Coating 

#### 2.2.1. SAN/PANI/FLG

Table 2 demonstrates materials employed for SAN/PANI/FLG coating.

##### Preparation of Polyaniline

Chemical oxidation polymerization technique was used to prepare conductive Polyaniline (PANI). 6 g of aniline monomer was mixed in 100 mL deionized water using a vessel fixed with a stirrer and equipped with ice bath for low temperature arrangement. Conductivity of the solution was improved by adding 1 M solution of HCl to obtain aniline hydrochloride; a continuous stirring was ensured during addition. As a surfactant, 1 M solution of Sodium Dodecyl Sulfate (SDS) was added. To start polymerization, 1.2 g APS (Ammonium per sulfate) was dissolved as initiator in a 20 g of deionized water. Gradual addition of the above solution was facilitated using a stirring mechanism while the low temperature (0 °C) was maintained with the help of an ice bath; the mixing continued for one hour. The stirring continued for one a half hour at room temperature. Methanol was used to facilitate precipitation followed by washing using deionized water for multiple times. Final drying was performed in an oven at 50 °C. A green colored PANI powder was the final product. Initially, methanol precipitation was performed. The polymer was then washed by using distilled water, this process of conditioning with distilled was repeated several times. After washing the precipitate, it was dried in an oven at 50 °C. A green colored PANI powder was produced as the final product from the above process.

##### Preparation of SAN/PANI/FLG Nano Composite

Thin film of nanocomposite coating was prepared through solution casting method. SAN polymer was added to 1,2-dichloroethane and then continuously stirred for 40 min in order to completely dissolve the constituents of the solution. Then PANI was slowly added; and the solution color turned dark green. Solution was probe sonicated and stirred overnight for proper dispersion of PANI. Once the SAN and PANI was completely dissolved, then an appropriate quantity of FLG was added. The solution was then subjected to probe sonication for 45 min to disperse the PANI and FLG. The acquired solution was kept on overnight stirring after which the nanocomposite was ready to coat on the substrate. Mechanical properties of the SAN/PANI/FLG thin film are shown in Table 3.

#### 2.2.2. ZnO/GO Coating

Ceramic coatings were developed on the metallic substrate in two steps: synthesis of (a) ZnO nanoparticles sol gel; (b) ZnO-GO composite sol.

(a) Synthesis of ZnO Nanoparticle Sol Gel

Zinc Acetate Dehydrate (ZnAcDH) was used as a precursor in the synthesis. Isopropanol alcohol was used as the solvent. Ethanol amine was used as stabilizer in a specific amount for the preparation of 0.4 M ZnO sol. It was then stirred until opaque solution turned into transparent one i.e., ZnAcDH was completely dissolved. The resultant solution was left for 24 h for aging. The ingredients are given in the Table 4.

(b) Synthesis of ZnO/GO Composite Sol

A solution containing 0.5 mg/1 mL of graphene oxide in 2-methoxyethanol was formulated and prepared. For proper dispersion of graphene oxide, sonication was performed for 45 min in ultrasonic water bath. Both graphene oxide and ZnO solutions were mixed together. The obtained ZnO nanoparticles had 13 nm to 22 nm size range.

### 2.3. Dip Coating 

Dip coating is a time efficient and most commercially adapted technique for coating purposes. Dip coating process involves five steps: (i) Immersion; (ii) Dwell time; (iii) Deposition; (iv) Drainage; and (v) Evaporation. Samples were dipped in the solution for 3 min. Dwell time was kept 0.5 min to achieve desired thickness. When the solution was properly deposited on the metal samples, the coated samples were then placed in an oven to drive off the solvent from the solution resulting in a meaningful deposition of thin films. Coating thicknesses were kept in a range from 5 to 7 μm, by controlling the process parameter e.g., dwell time etc. using the Landau–Levich equation. Coating thickness was measured by surface profilometry and further manually confirmed by micrometer screw gauge and Landau–Levich equation.

### 2.4. Electrochemical Testing

Gamry^®^ framework (Series G-750, Gamry Instruments, Inc., Warminster, PA, USA) equipped with DC corrosion testing as (Gamry Instruments, Inc.) EIS, was employed for all electrochemical measurements. EIS and Tafel scans were conducted by using three-electrode cell. The reference and counter electrodes in the cell were a Saturated Calomel Electrode (SCE) and a graphite rod respectfully. Metal samples of 4 cm × 4 cm dimensions were used. The surfaces were masked using epoxy leaving an exposed area of 3 cm × 3 cm. Two types of corrosive environments were used as electrolytes for investigation: (i) simulated seawater (pH slightly basic (7.9) and conductivity between ≥50 mS/cm); and (ii) a sample of produced water obtained from a local petroleum sector (pH 7.6) and bulk conductivity ~150 µS/cm).

### 2.5. Equivalent Circuit Modeling

**Figure 1:**
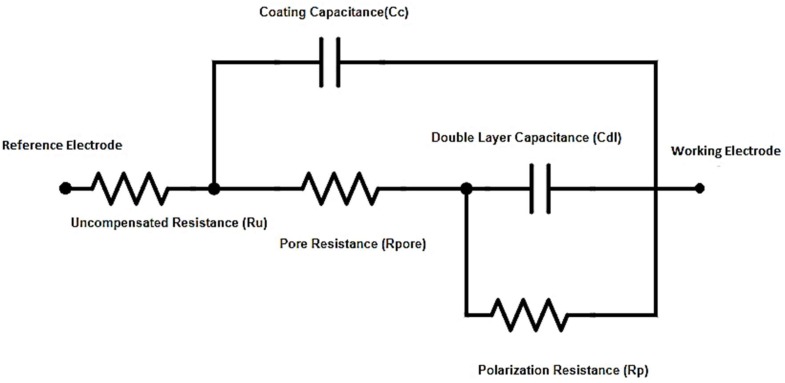
Equivalent cell circuit and EIS Input parameters (EIS-Input Parameters AC voltage: 10 mV; Initial frequency: 1 × 10^5^ Hz; Final frequency: 0.2 Hz; Point/decade: 10; Exposed area: 3 × 3 cm^2^; DC Potential Range: −250 mV to +250 mV vs. SCE).

Equivalent circuit model of the cell system with input parameters is shown in Figure 1. It was used for fitting the curves to obtain pore resistance and coating capacitance values of two nanocomposites systems using Reap2cpe model. The determined values are shown in the Table 5.

### 2.6. Surface Morphology

The surface morphology of the samples was studied before and after the corrosion test to observe the damage and deterioration caused by the corrosive environments. This morphological study was conducted by using Scanning Electron Microscope (JOEL JSM-6490A, JEOL USA, INC., Peabody, MA, USA).

## 3. Results and Discussion

### 3.1. Electrochemical Impedance Spectroscopy (EIS) in Seawater

**Figure 2:**
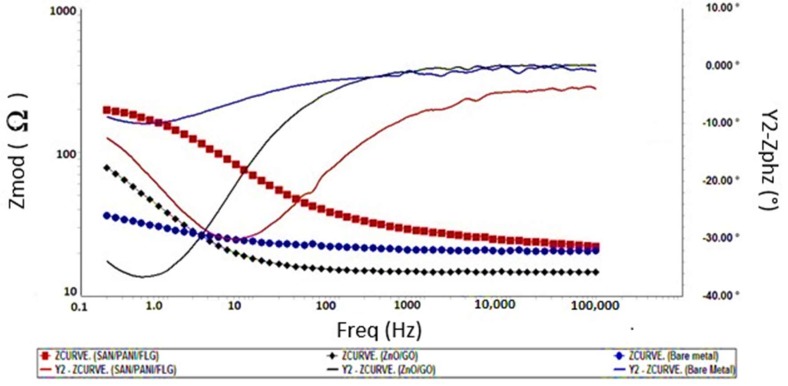
Bode Plots-bare and coated samples in seawater.

Seawater was used as a corrosive medium to test the coated samples by using EIS and Tafel scan and compared their corrosion resistance with bare metal sample. It is shown in the Figure 2 that the impedance value of bare metal approached 50 Ω which was the indication of the low resistance of the metal sample against corrosion. Among the coating systems, SAN/PANI/FLG coating has shown higher impedance value of 250 Ω whereas impedance of ZnO/GO coating was approximately 100 Ω indicating the higher protection capability of SAN/PANI/FLG coating in seawater compared with ZnO/GO system.

The phase shift difference in bare metal and the coated metal is also superimposed in Figure 2. The phase shift of ZnO/GO was found higher than SAN/PANI/FLG coating revealing a better capacitive behavior of ZnO/GO. It could be aligned to the greater surface activity of nano-ZnO that improved the absorption of water on its surface that increased the density of coating and blocked the passages of electrolyte through the coating, thereby improving the barrier protection against corrosion [21,22,23]. Mostafaei [23] reported similar work and studied the effect of Polyaniline and ZnO on corrosion protection. It was reported that the barrier against the corrosion of Epoxy/PANI/ZnO was 3 times higher by magnitude than Epoxy/PANI coating, and was 4 times higher than Epoxy coating [23].

**Figure 3:**
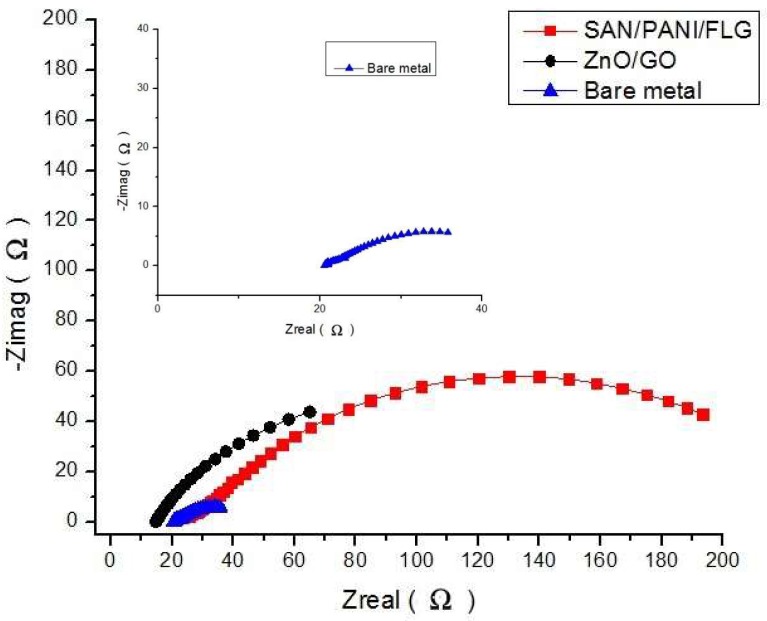
Nyquist Plots of bare and coated samples seawater.

Figure 3 displays Nyquist plots which appeared to agree with the Bode plots. SAN/PANI/FLG coated samples exhibited higher impedance value and thus higher corrosion resistance than ZnO/GO coated samples. Values of EIS parameters i.e., pore resistance (R_pore_), coating capacitance (C_c_), uncompensated/solution resistance (R_u_ or R_soln_), polarization resistance (R_p_ or R_corr_) and double layer capacitance (C_dl_ or C_corr_) obtained by fitting the respective curves through Reap2cpe (Rapid Electrochemical Assessment of Paint (REAP), Constant Phase Element (CPE)) model are shown as below in Table 5. 

The EIS data given in Table 5 was found helpful in determining the performance of coatings in corrosive environment. As shown in Table 5, SAN/PANI/FLG had higher pore resistance (R_pore_) than ZnO/GO coating indicating better electrochemical stability and corrosion resistance of the polymeric nanocomposite in seawater environment as compared to ceramic base nanocomposite system. The higher value of pore resistance can be attributed to reduced formation of pores in the coating, restricting the ability of the electrolyte to reach the metal’s surface, thereby decreasing the corrosion [15,16,17,18,19,20]; pore resistance parameters are also important for defining the chemical stability of coating in corrosive environment.

Coating capacitance of SAN/PANI/FLG system was found higher compared to ZnO/GO coating in seawater. It shows that the SAN/PANI/FLG has better water uptake and dielectric properties than ZnO/GO. The diffusion of electrolyte leads to diffusion of corrosive ions through thin film that expectedly affects the coating capacitance. Coating capacitance usually increases significantly with the increase of defects in the coating, when exposed to corrosive environment. This behavior was noticed in a few cases after immersion for short durations in aggressive environment, the value of C_c_ initially increased and then decreased gradually, presumably, a deposition of corrosion residues in pinholes and defects takes place, resulting in blocking the diffusion of corrosive species and H_2_O molecules into the coating [8].

### 3.2. DC Corrosion Testing: Tafel Scan in Seawater

**Figure 4:**
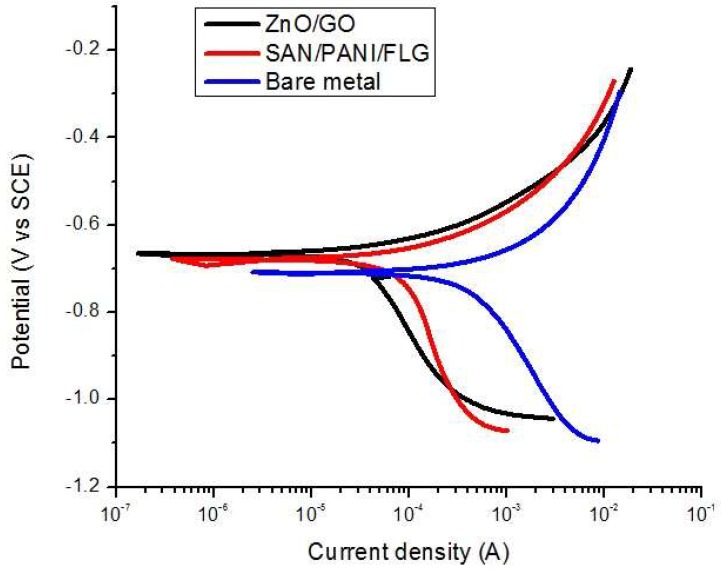
’E-log i’ curves-bare and coated samples in seawater.

Corrosion rate data from DC corrosion testing is given in Table 6 and the ‘E-log i’ curves are shown in Figure 4. It was revealed that the corrosion of bare metal was reduced up to 85% by SAN/PANI/FLG nanocomposite coating on the metal, whereas ZnO/GO coating suppressed the corrosion rate up to 75% of the bare metal as shown in the Table 6. As mentioned corrosion potential was also shifted to more positive directions for coated metal indicating improvement in corrosion resistance due to coatings [23,24,25].

Polyaniline shifts the potential to the noble directions which is presumed to generation of passive oxide layer providing an alternative conducting path for the electron flow. PANI passivates the pinholes through iron oxide on metal substrate surface. Polyaniline transfers the electron transport from metal surface to the outer surface of primer. Generally, in the process of corrosion protection, the release of acid dopant (camphorsulphonic acid) results in polyaniline leucosalt (PANI-LS) due to the reduction of polyaniline-emeraldine salt. The formation of a passive iron ions film within the defect sites is facilitated by these sulphonic ions. PANI-LS are re-oxidized by dissolved oxygen to PANI-ES. In other words, it can be assumed that at first PANI gets the ions that are released in the corrosion process of iron and gets doped. Once it gets doped, it releases the dopant ions which create the passive layer on the interface of metal and coating [25,26,27]. PANI functions as self-healing coating and this cyclic process helps in effective corrosion protection [25,26,27]. Wei-Kang Lu [11] reported the effect of polyaniline in protecting mild steel and shifting the corrosion potential to positive direction. This protection capability of Polyaniline to generate a passive oxide layer on the surface of the substrate has been discussed [11].

Corrosion protection of graphene is linked to several facts. It is hydrophobic due to which it resists hydrogen bonding with water while in parallel it has ability to prevent oxidation of metal. Its nano-sized structural defects lead to limited passivation. Moreover, it suppresses ion conduction preventing the formation of galvanic cells resulting in better protection properties [2,5].

The obtained value of the desired parameters confirmed better protection ability of SAN/PANI/FLG system due to lower value of corrosion rate as compared to that of ZnO/GO system.

### 3.3. Surface Morphology in Seawater

**Figure 5:**
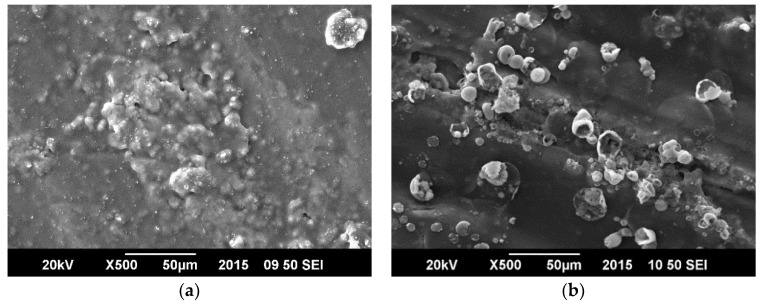
SEM micrographs of SAN/PANI/FLG coated samples in seawater. (**a**) Before corrosion test; (**b**) After corrosion test.

**Figure 6:**
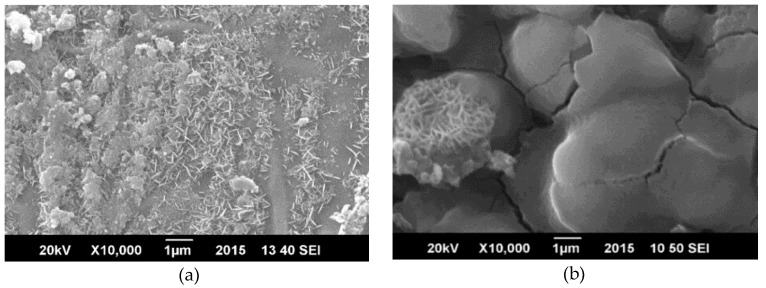
SEM micrographs-ZnO/GO coated samples in seawater (**a**) Before corrosion test; (**b**) After corrosion test.

Figure 5 shows that the surface morphology of the coating as observed under Scanning Electron Microscopy (SEM); the surface was altered by corrosion, but no significant cracks were developed in the coating indicating that the polymeric based coating afforded better protection properties. Figure 6 displays damage to ZnO/GO coating after the tests in which morphology was significantly changed by the cracks which are produced in the process of protection.

### 3.4. EIS Measurements in Crude Oil Produced Water 

**Figure 7:**
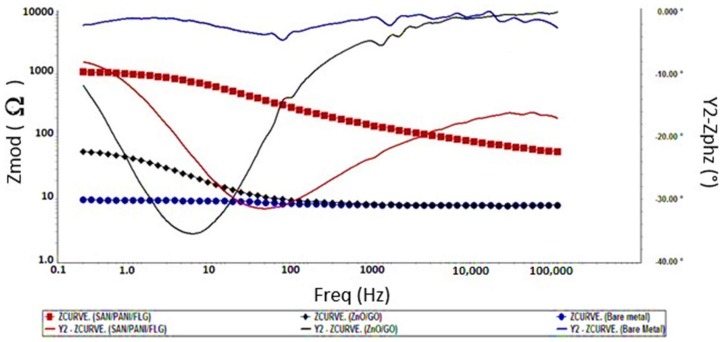
Comparison of Bode Plots (crude oil produced water).

Crude oil produced water holds various types of compounds and hydrocarbons along with impurities, which are aggressive and potentially harmful towards corrosion. EIS and DC corrosion tests were performed for bare metal and, SAN/PANI/FLG and ZnO/GO coated samples. Figure 7 displays Bode plots for bare metal, SAN/PANI/FLG coating and ZnO/GO coating. The graphs exhibit variation in impedance values of various systems and highlights phase shifts. SAN/PANI/FLG performed much better in this environment and offered better protection as compared to that of ZnO/GO and bare metal.

Relatively higher value of impedance approaching to 1000 Ω of SAN/PANI/FLG in produced water was noticed, whereas, the impedance of ZnO/GO was approximately 90 Ω. Likewise, the behavior in seawater environment, ZnO/GO also demonstrated a higher value of phase shift compared to SAN/PANI/FLG coating when exposed to produced water. This indicated its better capacitive and dielectric properties. However, being chemically unstable in this environment, it could not offer any significant resistance against corrosion [28,29].

**Figure 8:**
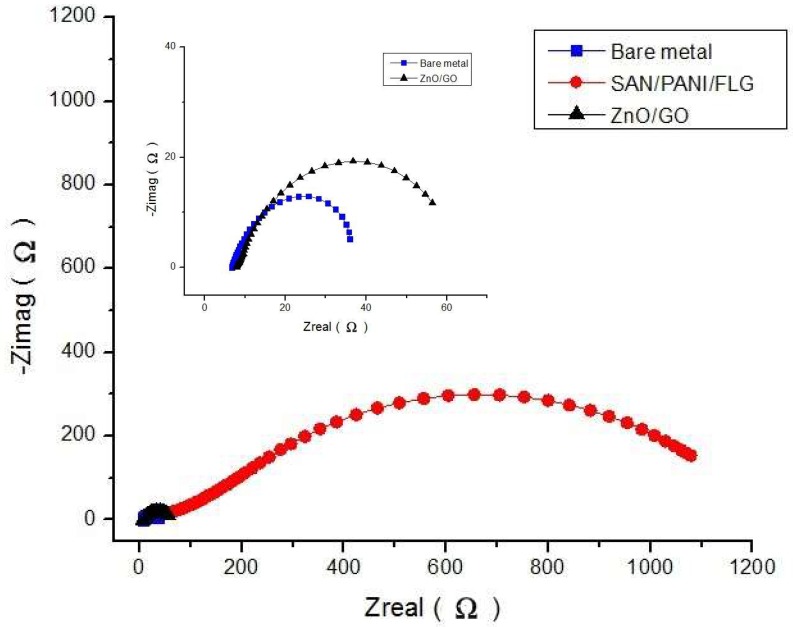
Comparison of Nyquist Plot (crude oil produced water).

As evident from Figure 8, Nyquist plots verified relatively higher corrosion resistance of SAN/PANI/FLG system in crude oil produced water compared with its ceramic counterpart. Significantly higher values of impedance for SAN/PANI/FLG system were observed skewing the other two systems. The Nyquist plots showed a few suppressed semicircles representing a capacitance which is slightly deviating from ideal behavior [30]. Generally, the Nyquist plots of all coatings appeared to be semicircles with their centers on the *x*-axis (impedance), it would appear that the characteristics of the system influences the suppressed semicircles. It can also be attributed to few inhomogeneous features of the system such as non-uniform distribution of various surface properties at various locations over the electrode surface [26,31,32].

Values of R_pore_ and C_c_ are mentioned in Table 7, which displays a significant difference in the R_pore_ values for SAN/PANI/FLG and ZnO/GO coatings. This indicates a better electrochemical stability and corrosion resistance capability of the polymeric nanocomposite system in produced water as compared to ceramic nanocomposite coating. 

Coating capacitance of ZnO/GO was observed to be slightly higher than SAN/PANI/FLG coating indicating larger water absorption and dielectric nature of ceramic nanocomposite than the polymeric nanocomposite [8].

### 3.5. DC Corrosion Testing-Tafel Scan in Crude Oil Produced Water

**Figure 9:**
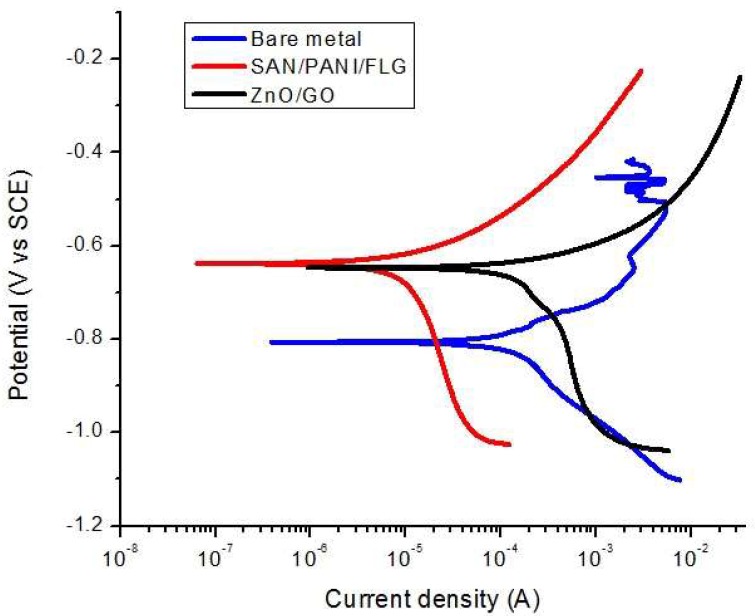
’E-log i’ curves-bare and coated samples in (crude oil produced water).

Tafel scan results validate EIS data regarding the performance of the nanocomposites coatings in produced water. Both coatings exhibited improved corrosion resistance compared to uncoated metal. Corrosion resistance of SAN/PANI/FLG coating was observed to be significantly better compared to ZnO/GO coating, as displayed in Figure 9, the data is summarized in Table 8. 

As evident from Table 8 and Figure 9, ZnO/GO system did not prove to be protecting and the corrosion rate was found to be very close to bare metal. The SAN/PANI/FLG system, however, exhibited about 27 times reduction in the rate of corrosion compared with bare iron metal. Both the nanocomposites coatings, however, shifted the E_corr_ value towards positive direction. The potential values display a qualitative check for aggressiveness of the electrolyte towards the working electrode, whereas, the quantitative data is based on the current values. In Figure 9, the potentials of the two coated samples although were in close vicinity, however, there position in terms of current were quite distant, presumably due to breakdown of the coating in the electrolyte used.

### 3.6. Surface Morphology in Crude Oil Produced Water 

**Figure 10:**
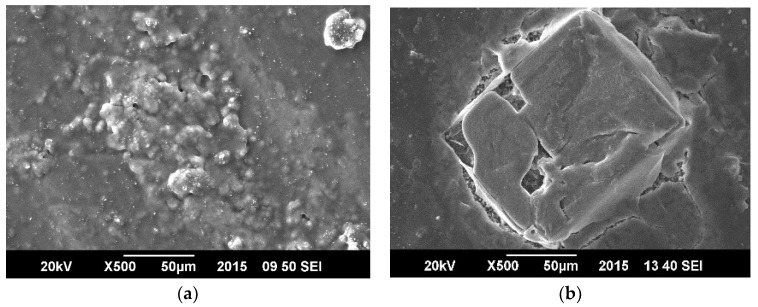
SEM image-SAN/PANI/FLG coated samples (produced water of crude oil). (**a**) Before corrosion test (**b**) After corrosion test.

Figure 10 displays SAN/PANI/FLG coated surface before and after corrosion testing in produced water the micrographs exhibit a partial degradation appearing in the form of micro-cracks in the coating after corrosion testing. The surface, however, did not appear to be damaged thoroughly, indicating relatively better resistance against corrosive attack by produced water. 

**Figure 11:**
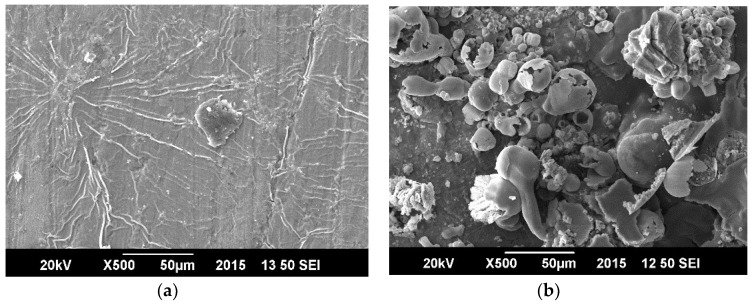
SEM image-ZnO/GO coated sample (produced water of crude oil) (**a**) Before corrosion test (**b**) After corrosion test.

Figure 11 shows morphology of ceramic nanocomposite coated surface. It was evident that the ceramic coating appeared to be significantly damaged after corrosion testing, indicating its weak resistance against corrosive attack of produced water.

## 4. Conclusions 

It was concluded that the corrosion protection of polymeric based SAN/PANI/FLG coating was found reasonably higher than ceramic based ZnO/GO coating in seawater as well as in produced water. SAN/PANI/FLG coating reduced the corrosion of bare metal up to 90% in seawater environment with the highest reported impedance value of 250 Ω, whereas, ZnO/GO suppressed the corrosion up to 75% having the impedance value of 100 Ω. 

In crude oil produced water, SAN/PANI/FLG coating proved to be significantly better corrosion resistant compared to ZnO/GO coating. SAN/PANI/FLG reduced the corrosion up to 95% with impedance value of 1000 Ω while ZnO/GO suppressed the corrosion up to 10% with the impedance value of 90 Ω. 

Both polymeric and ceramic based nanocomposites coatings demonstrated better protection against sea and produced water. However, the variation in protection levels of both polymeric and ceramic based nanocomposites coatings between the two environments can be seen. SAN/PANI/FLG coating performed better in terms of reducing corrosion of bare metal as compared to ZnO/GO coating. This was due to better resistance against pore formation and strong barrier properties against the electrolyte of SAN/PANI/FLG coating. 

## Figures and Tables

**Table 1:** Constituents proportion of ductile cast iron.

Element	Percentage (%)
Carbon	3.7
Silicon	2.6
Sulfur	0.3
Manganese	0.2
Iron	Balance

**Table 2:** Ingredients for SAN/PANI/FLG coating.

Material	wt.%	Manufacturer
SAN	90	ERKOL (Mohegan Lake, NY, USA)
Polyaniline	10	Prepared in lab
Few layer graphene (5–8 layers) (2 Micrometer sheet size)	0.1	I. Janowska

**Table 3:** Mechanical Properties of SAN/PANI/FLG thin film.

Mechanical Properties	Quantity
UTS of SAN/PANI/FLG thin film	26.03 MPa
Elastic Modulus (E)	1.37 GPa
Strain%-Strain at break	2.6%

**Table 4:** Ingredients for ZnO/GO coating.

Material	Quantity
Zinc Acetate Dihydrate	4.42 g
Ethanol amine	50 mL
Isopropanol Alcohol	1.207 mL

**Table 5:** Reap2cpe fitting values of coated samples in sea water.

SAN/PANI/FLG		ZnO/GO	
R_pore_	82.79 Ω	R_pore_	6.648 Ω
C_c_	2.105 × 10^−3^ F	C_c_	1.354 × 10^−3^ F
R_soln_	11.53 Ω	R_soln_	8.150 Ω
R_corr_	663.1 × 10^9^ Ω	R_corr_	196.8 Ω
C_corr_	33.90 × 10^−3^ F	C_corr_	8.978 × 10^−3^ F

**Table 6:** Corrosion rate (C.R) data from Tafel scan.

Corrosion Rate and Potential	Bare Metal	SAN/PANI/FLG	ZnO/GO
C.R (mpy)	19.56	2.514	5.827
E_corr_ (mV)	−708.1	−677.5	−665.1

**Table 7:** Reap2cpe fitting values of coated samples in produced water.

SAN/PANI/FLG		ZnO/GO	
R_pore_	114.0 Ω	R_pore_	2.260 Ω
C_c_	55.01 × 10^−6^ F	C_c_	1.775 × 10^−3^ F
R_soln_	34.95 Ω	R_soln_	5.687 Ω
R_corr_	1.125 × 10^3^ Ω	R_corr_	67.01 Ω
C_corr_	83.08 × 10^−6^ F	C_corr_	4.641 × 10^−3^ F

**Table 8:** Corrosion rate data from Tafel scan in produced water.

Corrosion Rate and Potential	Bare Metal	SAN/PANI/FLG	ZnO/GO
C.R (mpy)	21.76	0.7867	20.82
E_corr_ (mV)	−805.8	−637.4	−629.1
